# Single-cell RNA-sequencing profiles reveal the developmental landscape of the *Manihot esculenta* Crantz leaves

**DOI:** 10.1093/plphys/kiad500

**Published:** 2023-09-14

**Authors:** Yuwei Zang, Yechun Pei, Xinli Cong, Fangfang Ran, Liangwang Liu, Changyi Wang, Dayong Wang, Yi Min

**Affiliations:** Department of Biotechnology, School of Life Sciences, Hainan University, Haikou, Hainan 570228, China; Department of Biotechnology, School of Life Sciences, Hainan University, Haikou, Hainan 570228, China; Laboratory of Biopharmaceuticals and Molecular Pharmacology, School of Pharmaceutical Sciences, Hainan University, Haikou, Hainan 570228, China; Department of Biotechnology, School of Life Sciences, Hainan University, Haikou, Hainan 570228, China; Laboratory of Biopharmaceuticals and Molecular Pharmacology, School of Pharmaceutical Sciences, Hainan University, Haikou, Hainan 570228, China; Department of Biotechnology, School of Life Sciences, Hainan University, Haikou, Hainan 570228, China; Department of Biotechnology, School of Life Sciences, Hainan University, Haikou, Hainan 570228, China; Department of Biotechnology, School of Life Sciences, Hainan University, Haikou, Hainan 570228, China; Laboratory of Biopharmaceuticals and Molecular Pharmacology, School of Pharmaceutical Sciences, Hainan University, Haikou, Hainan 570228, China; Department of Biotechnology, School of Life Sciences, Hainan University, Haikou, Hainan 570228, China

## Abstract

Cassava (*Manihot esculenta* Crantz) is an important crop with a high photosynthetic rate and high yield. It is classified as a C3–C4 plant based on its photosynthetic and structural characteristics. To investigate the structural and photosynthetic characteristics of cassava leaves at the cellular level, we created a single-cell transcriptome atlas of cassava leaves. A total of 11,177 high-quality leaf cells were divided into 15 cell clusters. Based on leaf cell marker genes, we identified 3 major tissues of cassava leaves, which were mesophyll, epidermis, and vascular tissue, and analyzed their distinctive properties and metabolic activity. To supplement the genes for identifying the types of leaf cells, we screened 120 candidate marker genes. We constructed a leaf cell development trajectory map and discovered 6 genes related to cell differentiation fate. The structural and photosynthetic properties of cassava leaves analyzed at the single cellular level provide a theoretical foundation for further enhancing cassava yield and nutrition.

## Teaser

A single-cell fine map of cassava leaves reveals various types of cell characteristics and functions.

## Introduction

In addition to being an important food crop in tropical and subtropical areas, cassava (*Manihot esculenta* Crantz), a perennial woody shrub of the Euphorbiaceae family, is also a source of renewable energy (Obata et al. 2020). The primary site of starch storage is the cassava root, and the leaves may act as a metabolic switch to initiate or control the growth of storage roots (Mitprasat et al. 2011). Additionally, cassava leaves are a good source of protein, minerals, and vitamins, making them a valuable addition to human and animal diets (Bradbury and Denton 2014; Letif and Muller 2015; Jiwuba et al. 2021). Angiosperms can be classified as C3 plants, C4 plants, or CAM plants based on different carbon assimilation pathways. Cassava differs from C3 plants in that it has a high rate of photosynthetic activity and a low rate of light respiration (De Souza et al. 2017). Additionally, the C4 pathway's essential enzymes phosphoenolpyruvate carboxylase and phosphoglycolate phosphatase are active in cassava, but it lacks the Kranz rosette structure that is present in the leaves of C4 plants (El-Sharkawy 2004, 2016). Therefore, cassava is classified as a C3–C4 plant (Wang et al. 2020). A theoretical foundation for further enhancing cassava yield and nutrition can be found by investigating the photosynthesis of cassava leaves at the cellular level.

The leaves of dicotyledonous plants are primarily composed of epidermis, mesophyll, and vascular tissues (Ma et al. 2012). Pavement cells (PCs), stomata, and trichomes are 3 different types of plant epidermal cells whose primary function is to shield plants from pathogens and environmental stress (Takada and Iida 2014; da Silva et al. 2019). Mesophyll cells differentiate into palisade mesophyll cells and spongy mesophyll cells. Palisade mesophyll cells are closely arranged and close to the epidermal layer of the plant's dorsal axis, while spongy mesophyll cells have different shapes and are located below palisade mesophyll cells (Silva et al. 2017). Vascular tissue is an essential conducting tissue in plant leaves. It is made up of xylem, phloem, and bundle sheath (BS), which transport water and carbohydrates through tracheary elements (TEs), sieve elements (SEs), and companion cells (CCs) (Dinneny and Yanofsky 2004; Bomfim et al. 2011; Graciano-Ribeiro and Nassar 2012). A thorough understanding of the distribution of cell types in leaves will aid in the further elucidation of cell functions and connections.

Single-cell RNA sequencing (scRNA-seq) is a method that employs high-throughput sequencing of isolated single cells to obtain all gene expression information in a cell (Tang et al. 2011). This method can be used to identify different cell types, analyze the temporal and spatial development of different cells, and reveal the heterogeneity of gene expression between cells (Shojaee et al. 2021; Tenorio Berrio et al. 2022). In recent years, the application of scRNA-seq in plants has developed, not only in model plants such as *Arabidopsis* (*Arabidopsis thaliana*), rice (*Oryza sativa* L.), and maize (*Zea mays* L.) but also in nonmodel plants including poplar (*Populus alba* var. *pyramidalis*) and cotton (*Gossypium hirsutum* L.) (Chen, Lv, et al. 2021; Chen, Tong, et al. 2021; Han et al. 2017; Hey et al. 2017; Shahan et al. 2021; Apelt et al. 2022; Liu et al. 2022). However, scRNA-seq has not been applied to C3–C4 plants such as cassava. This study can provide important value for the evolution of C3 plants to C4 plants.

This study utilized the scRNA-seq method to construct the scRNA-seq expression landscape of the cassava leaf at the single-cell level. By using marker genes, we were able to determine the various cell types that were present in cassava leaves. We then examined the characteristics and functions of the various cell populations. Then, using the differential genes of different cellular populations, we mined for previously unreported marker genes. Finally, we examined the growth and development characteristics of cassava leaves using pseudotime analysis. Our study demonstrates the applicability of scRNA-seq technology to shrub plants and C3–C4 plants, offers a benchmark for the creation of nonmodel plant cell atlases, and establishes a theoretical foundation for future improvements in cassava quality.

## Results

### Distinct cellular subpopulations revealed by single-cell transcriptomics of leaves

**Figure 1.**
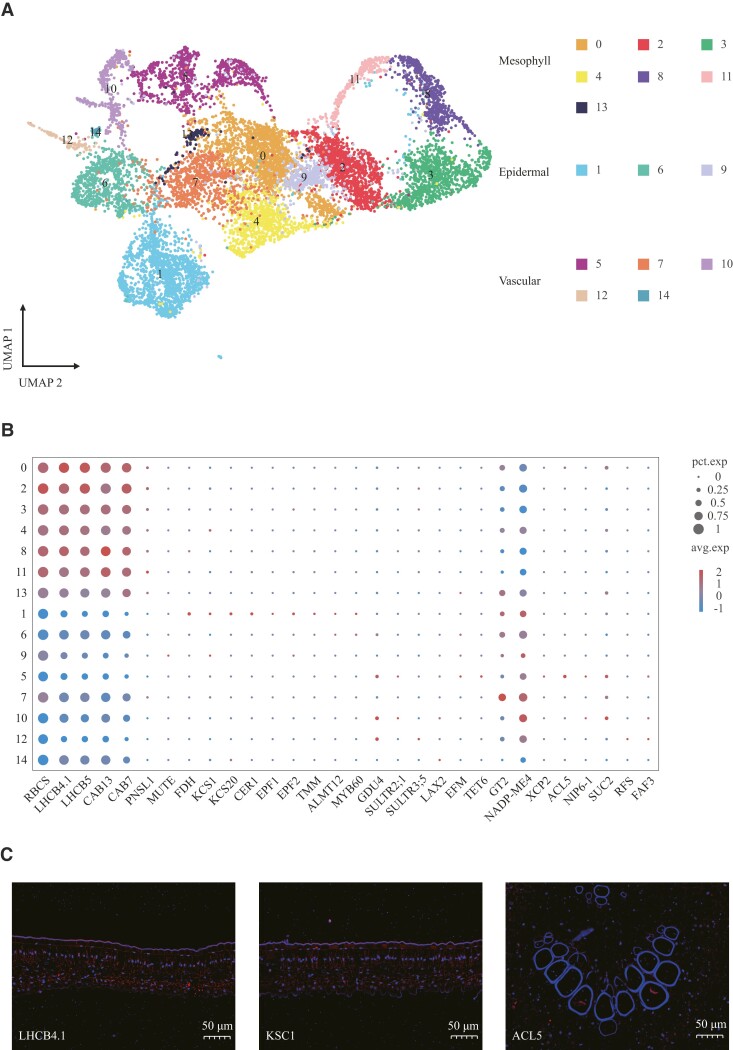
Cassava leaf transcriptomic landscape profiled by scRNA-seq and final annotation. **A)** Unsupervised clustering distinguished 15 preliminary cell populations (#0 to #14) visualized in a color-coded UMAP plot. In a UMAP plot, the distance between cells is associated with the transcriptomic divergence. Different colors represent different cell clusters. **B)** Dot plot showing the normalized expression of the marker genes used to annotate each tissue. The size of the dots represents the percentage of cells in the clusters that express the gene. The colors represent relative gene expression (blue = low; red = maximal expression of the gene). Detailed information on selected genes is given in Supplemental Table S3. **C)** In situ hybridization on different leaf slices from the same leaf sample for marker gene localization. Blue fluorescence corresponds to DAPI binding locations, while red fluorescence indicates the binding sites of gene probes containing Cys (bars = 50 *µ*m).

To investigate cellular heterogeneity during leaf development, we isolated plant cells from young leaves of the cassava variety “South China 8” (SC8). Following cell wall digestion, we successfully generated 3.03 × 10^6^ protoplasts, which exhibited high cell viability of 91% (Supplemental Table S1) as determined by trypan blue staining. Utilizing Chromium technology (10X Genomics), we performed scRNA-seq on the protoplasts to construct individual libraries. After thorough data filtering, we retained 11,177 high-quality protoplasts, with an average (median) expression of 2,536 genes (Supplemental Table S1 and Figs. S1 and S2). Employing the Louvain clustering method for unsupervised clustering, we then visualized the local similarity and global structure of cell populations using uniform manifold approximation and projection (UMAP) and *t*-distributed stochastic neighbor-embedding (t-SNE) projections. This unbiased approach identified 15 distinct cell populations labeled #0 to #14 (Fig. 1A; Supplemental Fig. S3 and Table S2).

### Elucidation of leaf tissue composition using scRNA-seq

Using the homologous sequences of *Arabidopsis* marker genes, we can identify most of the main cell types in cassava leaves by a single-cell transcriptome atlas (Fig. 1B; Supplemental Table S3). Firstly, we described 3 main tissues of leaves: mesophyll, vascular system, and epidermis. Clusters #0, #2, #3, #4, #8, #11, and #13 were defined as mesophyll cells by the mesophyll marker genes *chlorophyll a-b binding protein CP29.1* (*LHCB4.1*), *chlorophyll a-b binding protein CP26* (*LHCB5*), and *ribulose bisphosphate carboxylase small chain* (*RBCS*) (Supplemental Table S4) (Feng et al. 2014; Chen et al. 2018). These clusters had high photosynthetic activity and photosynthetic genes (Supplemental Table S5), such as the gene of the *photosystem I reaction center subunit IV A* (*PSAE1*) and *oxygen-evolving enhancer protein 2* (*PSBP*) (Tsai et al. 2017; Krieger-Liszkay et al. 2020). Clusters #1 and #9 were epidermal cells because they specifically expressed genes related to cuticular wax and suberin biosynthesis, such as *3-ketoacyl-CoA synthase 1* (*KCS1*), *3-ketoacyl-CoA synthase 10* (*FDH*), and *3-ketoacyl-CoA synthase 20* (*KCS20*) (Todd et al. 1999; Bird and Gray 2003; Lee et al. 2009). *GLUTAMINE DUMPER 4* (*GDU4*) is a probable subunit of an amino acid transporter involved in the regulation of amino acid metabolism and is expressed in the vascular tissue of leaves (Guerra et al. 2013). The gene was expressed in Clusters #5, #7, #10, #12, and #14, indicating that these clusters were vascular populations. After the expression analysis of classical marker genes, Cluster #6 was still not identified. Cluster #6 and Cluster #1 had the same gene ontology (GO) functional enrichment pattern, so Cluster #6 was defined as epidermal cells (Supplemental Fig. S4). The results of in situ hybridization were consistent with the types of cell subsets we identified, and the single-cell transcriptome data were used for further analysis (Fig. 1C; Supplemental Figs. S5 and S6).

### Characterization of the epidermal cell populations

**Figure 2.**
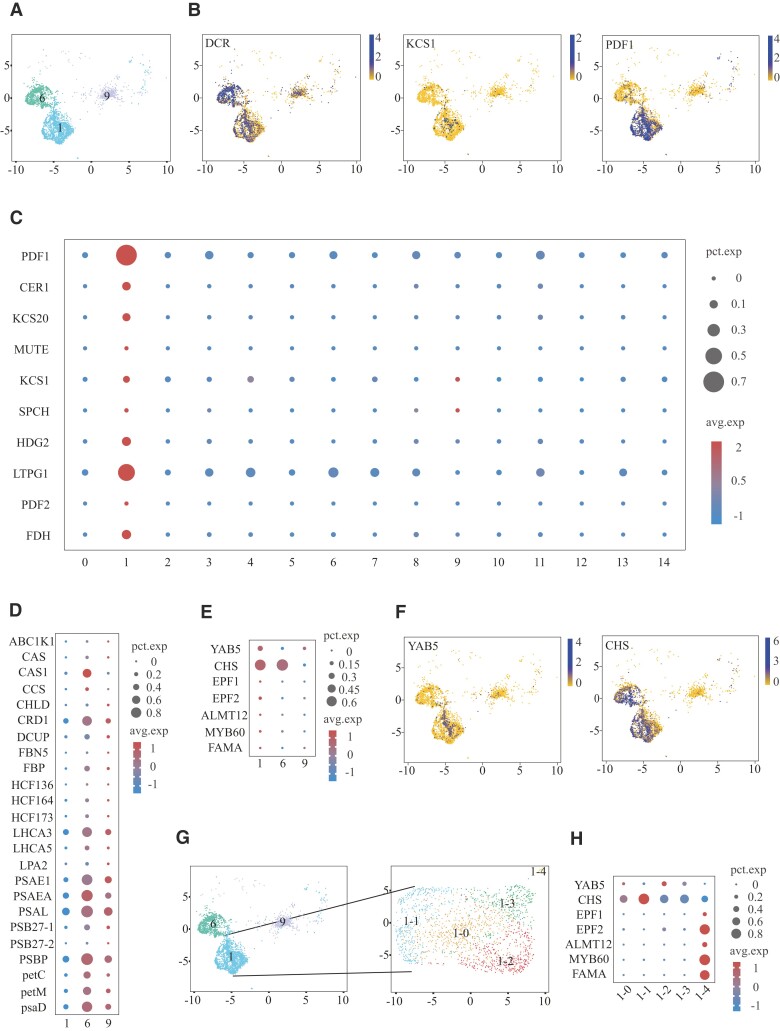
Identification of epidermal cell populations. **A)** Highlights of the epidermal cell populations in the UMAP plot. Different colors represent different cell clusters. **B)** UMAP plot with the normalized expression of the epidermal cell marker genes (blue = high; yellow = low). **C)** Dotplot with epidermal marker genes of each cell cluster. **D)** Dotplot with photosynthesis-related genes of epidermal cell populations. **E)** Dotplot with abaxial epidermal cells, adaxial epidermal cells, and guard cells marker genes of epidermal cell populations. **F)** UMAP plot with the normalized expression of the abaxial epidermal cells and adaxial epidermal cells marker genes (blue = high; yellow = low). **G)** Highlights of Cluster #1 cell subgroup in the UMAP plot. Different colors represent different cell clusters. **H)** Dotplot with abaxial epidermal cells, adaxial epidermal cells, and guard cells marker genes of Cluster #1 cell subgroup.

The epidermis consists of 3 types of cells: PCs, guard cells (GCs), and trichome cells. In our study, no cells expressing trichome cell marker genes (*GLABROUS/GLABRA1 GL1*, *GL2*, and *GL3*) were found. *KCS1* and *KCS20* are involved in elongation of C22 fatty acids for cuticular biosynthesis, which is a process occurring in PCs. These genes are expressed in Clusters #1, #6, and #9 (Fig. 2, A to C; Supplemental Fig. S7). Then, we compared Clusters #1, #6, and #9 by differential expression analysis. The YABBY family genes that determine the fate of abaxial cells, such as *axial regulator YABBY 5* (*YAB5*) (Yamada et al. 2011), were found to be partially expressed in Clusters #1 and #9 (Fig. 2, E and F). *Chalcone synthase* (*CHS*) was expressed in Clusters #1 and #6 (Fig. 2, E and F), which are mainly involved in flavonoid biosynthesis and are expressed in the adaxial epidermis (Dong et al. 2022). GO enrichment analysis of the first 100 differentially expressed genes (DEGs) in Clusters #1, #6, and #9 showed that Clusters #6 and #9 had many photosynthesis-related reactions (Supplemental Table S6). PSI-LHCI biogenesis-related genes such as *LHCA5* and *PSBP* were partially expressed in Clusters #6 and #9 (Fig. 2D). This indicates that some cells in Clusters #6 and #9 contain chloroplasts. Clusters #1 and #6 found different stimulus responses (Supplemental Table S6). The cells of Clusters #1 and #6 could receive external stimulus signals and transmit them to other cells. Moreover, GO enrichment analysis found that “response to salicylic acid,” “salicylic acid–mediated signaling pathway,” and “cellular response to salicylic acid stimulation” were enriched in Cluster #1 (Supplemental Table S6). This result indicated that salicylic acid was transported to epidermal cells and played an important role. We found that the cells in Cluster #1 were highly expressed *transcription factor FAMA* (*FAMA*) and *myb-related protein 306* (*MYB60*) (Rasouli et al. 2020), indicating that Cluster #1 contained guard cells (Fig. 2E). Since 3 types of cells were identified in Cluster #1, cell regrouping analysis was performed on Cluster #1. Cluster #1 was divided into 5 subgroups, named Clusters #1-0, #1-1, #1-2, #1-3, and #1-4 (Fig. 2, G and H). Clusters #1-2 and #1-3 highly expressed *YAB5*, Clusters #1-0 and #1-1 highly expressed *CHS*, and Cluster #1-4 highly expressed guard cell marker genes (Fig. 2, G and H). Therefore, we defined Clusters #1-2 and #1-3 as abaxial epidermal cells, Clusters #1-0 and #1-1 as adaxial epidermal cells, and Cluster #1-4 as guard cells.

### Identification of the mesophyll cell populations

**Figure 3.**
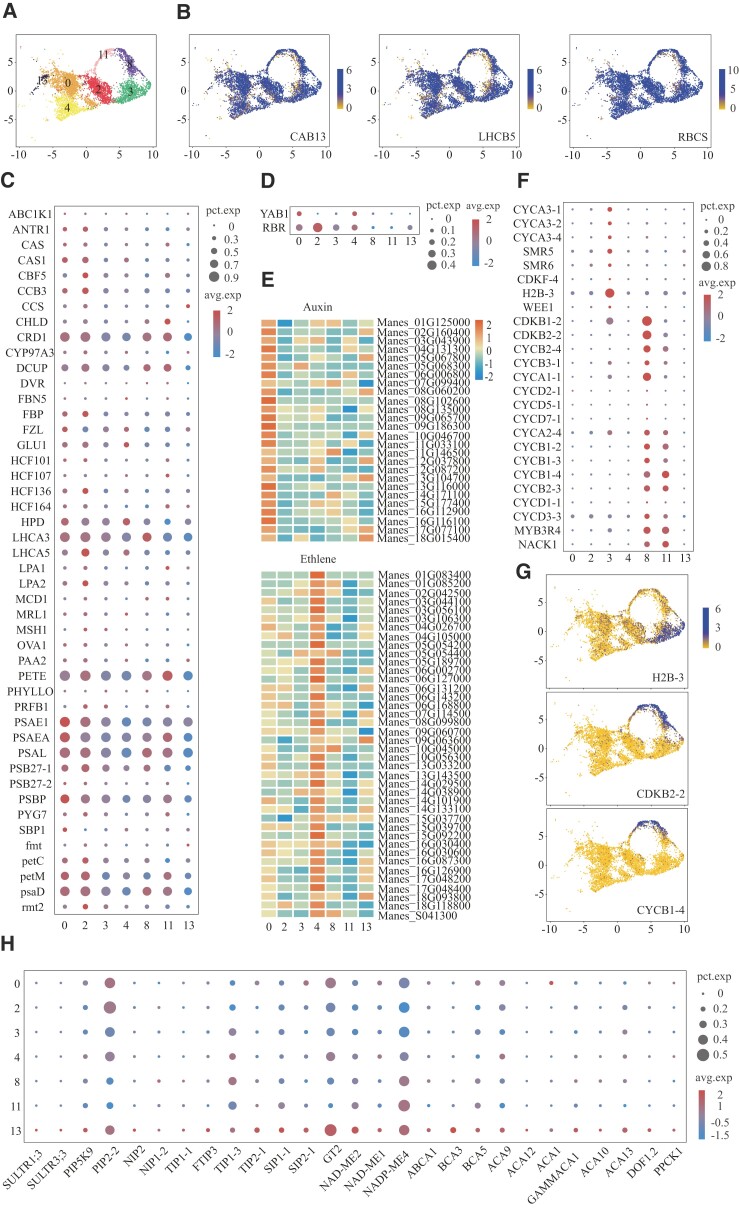
Identification of the mesophyll cell populations. **A)** Highlights of the mesophyll clusters in the UMAP plot. Different colors represent different cell clusters. **B)** UMAP plot with the normalized expression of the mesophyll cell marker gene (blue = high; yellow = low). **C)** Dotplot with photosynthesis-related genes of mesophyll cell populations. **D)** Dotplot with sponge mesophyll cells and palisade mesophyll cells marker genes of mesophyll cell populations. **E)** Heatmap illustrating the expression level of hormone-related genes identified in this study. On the vertical axis, the hormone-specific markers from this data set. On the horizontal axis, the clusters from mesophyll cell populations. The colors represent the normalized expression of the gene (red = high; blue = low). **F)** Dotplot with marker genes of the G1/S and G2/M cell cycle phase. **G)** UMAP plot with the normalized expression of the cell cycle marker gene. **H)** Dotplot with substance transport genes between mesophyll cells and bundle sheath cells of mesophyll cell populations of mesophyll cell populations.

The mesophyll cells are embedded between the abaxial and adaxial epidermis, defined as all photosynthetic cells surrounded by these epidermis cells. According to the expression patterns of marker genes depicted in Fig. 1, Clusters #0, #2, #3, #4, #8, #11, and #13 were unequivocally classified as mesophyll cell populations (Fig. 3, A and B). Clusters #0, #2, and #4 display a prominent upregulation of photosynthesis-related genes, suggesting their predominant composition of typical photosynthetic cells commonly found in the mesophyll tissue (Fig. 3C). *YABBY 1* (*YAB1*) is a known marker gene of spongy mesophyll cells (Filyushin et al. 2018), and *retinoblastoma-related protein* (*RBR*) is a known marker gene of palisade mesophyll cells (Dorca-Fornell et al. 2013). Based on the expression of these genes, we determined that Clusters #0 and #4 were sponge mesophyll cells and Cluster #2 was palisade mesophyll cells (Fig. 3D). Then, we performed differential gene expression analysis and GO enrichment analysis of the top 100 differential genes in Clusters #0, #2, and #4 (Supplemental Table S7). The auxin-related functions were enriched in Cluster #0 alone (Fig. 3E; Supplemental Table S8). The expression of *protein transport inhibitor response 1* (*TIR1*) and *protein AUXIN SIGNALING F-BOX 2* (*AFB2*) indicated that the synthesized auxin was transported to the mesophyll cells for preservation (Zhang et al. 2014). In Cluster #4, the functions related to ethylene metabolism were enriched (Supplemental Table S9). Genes related to the ethylene signal transduction pathway were found to be expressed in Cluster #4 (Fig. 3E), indicating that this group of mesophyll cells can bind to ethylene and activate the ethylene signal transduction pathway, therefore promoting leaf growth. We found that these mesophyll cells may be classified into different clusters due to differences in hormone function. We found that Clusters #3, #8, and #11 enriched genes related to cell division, indicating that the cells in these clusters were in the cell division cycle stage (Fig. 3F; Supplemental Table S10). *Histone H2B.3* (*H2B-3*) is a gene assembled by chromatin tissue or nucleosome (Jiang et al. 2020). *Proliferating cell nuclear antigen* (*PCNA*) is a gene related to DNA biosynthesis and chain elongation (Egelkrout et al. 2002). We found that *H2B-3* and *PCNA* were highly expressed in Cluster #3 (Fig. 3, F and G). CDKB is a kind of plant-specific cyclin-dependent kinase (CDK), which is only transcribed at G2 and M stages (Czerednik et al. 2012). *CDKB1-2* and *cyclin-dependent kinase B2-2* (*CDKB2-2*) were found to be enriched in Cluster #8 but not expressed in Cluster #11 (Fig. 3, F and G; Supplemental Fig. S8). These results suggested that the mesophyll cells undergoing DNA synthesis and replication were Cluster #3, the mesophyll cells undergoing cell division material preparation in G2 phase were Cluster #11, and the mesophyll cells undergoing cell division were Cluster #8. On the UMAP plot, Cluster #13 was close to the vascular tissue, which may be mesophyll cells around the vascular tissue (Fig. 3H; Supplemental Table S11). *NAD-dependent kinase 2* (*NAD-ME2*) and *phosphoenolpyruvate carboxylase kinase 1* (*PPCK1*) can transport Asp and Ala between mesophyll cells and vascular BS cells (Monreal et al. 2010; Badia et al. 2017). *NAD-ME2* and *PPCK1* were specifically expressed in Cluster #13. And Cluster #13 enriched the material transport functions such as “fluid transport,” “protein transport,” “single-organism intracellular transport,” “cytoplasmic transport,” and “intracellular transport” (Supplemental Table S7).

### Main cell types of vascular tissue

**Figure 4.**
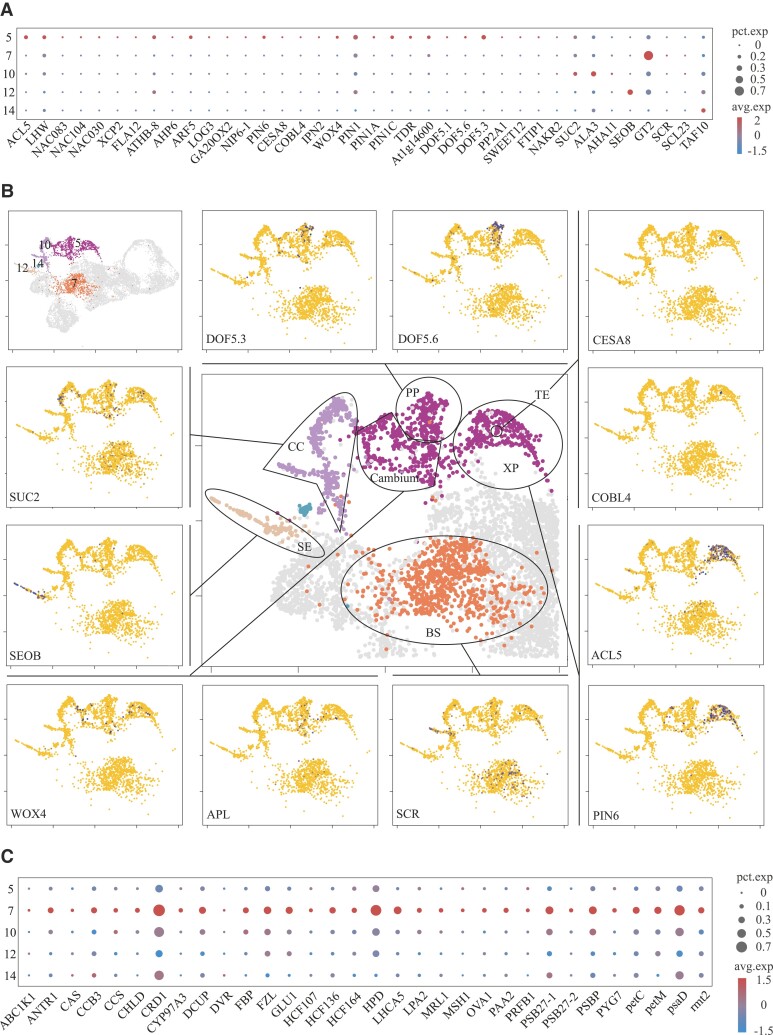
Identification of vascular cell populations. **A)** Dotplot with marker genes of vascular cell populations. **B)** On the top-left panel, the clusters classified as the vascular tissue are highlighted. The middle panel represents a close-up view of Clusters #5, #7, #10, #12, and #14 with indication of the subpopulations of the vascular tissue. These were identified based on the expression of the marker genes depicted in the surrounding panels. Different UMAP plots show normalized expression of the genes (blue = high; yellow = low). **C)** Dotplot with photosynthesis-related genes of vascular cell populations.

Vascular tissue is embedded in the mesophyll, which is responsible for bidirectional transport and distribution of molecules to target cells. Vascular tissue is composed of many different types of cell groups, such as xylem parenchyma (XP), phloem parenchyma (PP), phloem CCs, SEs, TEs, and BS. Our goal is to describe the main cell population of the vascular tissue. Through the expression of *dof zinc finger protein DOF5.6* (*DOF5.6*) (Guo et al. 2009), we defined some cells in vascular system Cluster #5 as PP (Fig. 4, A and B; Supplemental Fig. S9). SEs are tubular cells in the phloem, responsible for the long-distance transport of photosynthetic products and a variety of organic compounds in plants. *SIEVE ELEMENT OCCLUSION B* (*SEOB*) is a scaffold protein in the sieve tube (Jekat et al. 2013). We found that this gene was specifically expressed in the cells of Cluster #12, which was defined as SEs (Fig. 4, A and B). Cluster #10 cells were defined as CC (Fig. 4, A and B), and they expressed typical CC marker genes such as s*ucrose transport protein SUC2* (*SUC2*) and *protein SODIUM POTASSIUM ROOT DEFECTIVE 1* (*NAKR2*) (Tian et al. 2010; Wippel and Sauer 2012). The xylem was identified by the expression patterns of *thermospermine synthase ACAULIS5* (*ACL5*) and *auxin efflux carrier component 6* (*PIN6*) (Yoshimoto et al. 2012; Alabdallah et al. 2017). We defined the cells expressing these genes in Cluster #5 as XP (Fig. 4, A and B). A few TE differentiation markers, *cellulose synthase A catalytic subunit 8* (*CESA8*) and *COBRA-like protein 4* (*COBL4*) (Sato et al. 2010; Olins et al. 2018), were found in the Cluster #5 population, which were defined as TEs (Fig. 4, A and B). Using 2 cambium marker genes, *WUSCHEL-related homeobox 4* (*WOX4*) and *leucine-rich repeat receptor-like protein kinase TDR* (*TDR*) (Hirakawa et al. 2010; Suer et al. 2011), we defined Cluster #5 cells as cambium cells. (Fig. 4, A and B). The BS is a single-layer cell between mesophyll cells and vascular cells that is responsible for controlling the transport of metabolites and participating in photosynthesis. SCARECROW (SCR) and SCARECROW-LIKE PROTEIN 23 (SCL23) play a key role in the regulation of BS cell fate (Gao et al. 2014). We found that these genes are highly expressed in the #7 cluster, so we defined the #7 cluster as BS (Fig. 4C). Compared with other vascular tissue cells, Cluster #7 was enriched in photosynthesis-related functions. In Cluster #14, although the specific types of vascular tissue marker genes were not expressed in Cluster #14, GO functional analysis enriched for “divalent metal ion transport,” “fluid transport,” “proton transport,” “monosaccharide metabolic process,” and “hydrogen transport” (Supplemental Table S12). We believe that Cluster #14 belongs to vascular cells, but the specific type remains to be further studied.

### Identification of marker genes to unravel the leaf single-cell atlas

**Figure 5.**
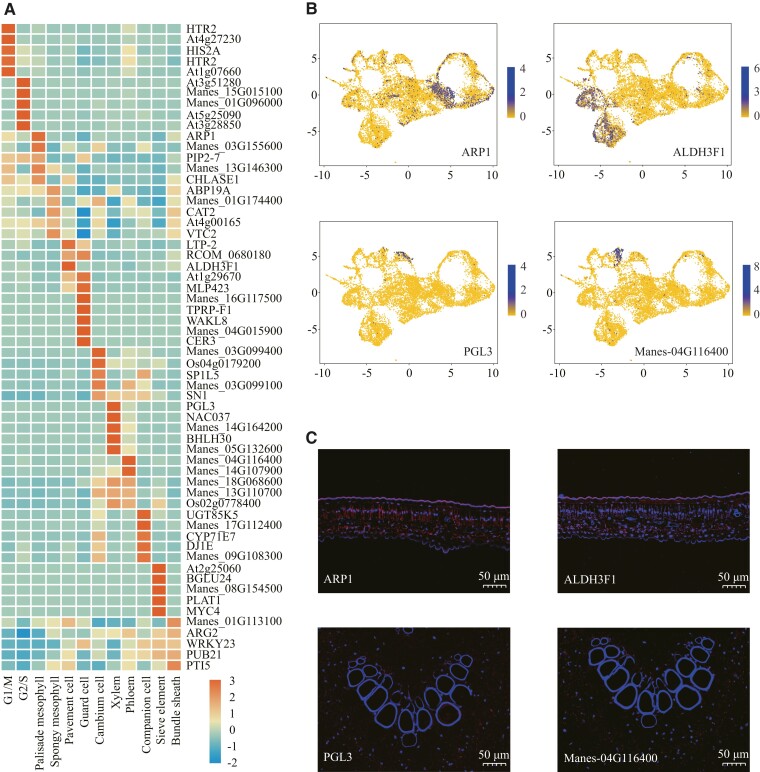
Identification of previously unreported marker genes within cell-type populations. **A)** Heatmap shows the top 5 DEGs (*P*-value ranking) in each subcluster (red = high; blue = low). **B)** The expression patterns of 8 previously unreported marker genes distributed in a UMAP plots (blue = high; yellow = low). **C)** In situ hybridization on additional leaf slices from the same leaf sample for marker gene localization. Blue fluorescence corresponds to DAPI binding locations, while red fluorescence indicates the binding sites of gene probes containing Cys (bars = 50 *µ*m).

We performed a functional analysis of the top 100 (*P*-value ranking) DEGs in each type of cell population, and the top 10 most relevant biological processes (BPs) were selected to functionally annotate each type of cell population (Table 1). In our study, G1/S phase cells were mainly involved in cell cycle DNA replication and DNA metabolism. G2/M phase cells were enriched for “microtubule-based process” and “mitotic cytokinetic process,” indicating that G2/M phase cells were undergoing cell division. Most of the functions of palisade mesophyll cells and sponge mesophyll cells were similar. The main difference is that palisade mesophyll cells were involved in protein modification and regulation, and sponge mesophyll cells were involved in gene expression and RNA modification. Epidermal cells were mainly involved in stress responses and responses to other substances, which was consistent with their protective role. In addition to the function of material transport, vascular tissue cells also had the function of responding to stress and mediating hormone-signaling pathways. We selected the top 10 genes with the highest expression levels in each type of cell population, a total of 120 genes, and described the expression profiles of the top 60 genes in the heatmap (Fig. 5A and Table 2; Supplemental Tables S13 and S14). We selected the most representative gene from each type of cell population for display in the UMAP map and in situ hybridization (Fig. 5, B and C). These identified DEGs will help to distinguish cell types in future scRNA-seq studies of plant leaves.

**Table 1.** Top 10 BP from GO terms obtained from the top 100 positively DEGs of 15 leaf populations

Cell population	Go enrichment BP
Cell cycle G1/S	Nucleosome organization, gene expression, single organism process, RNA modification, regulation of gene expression, cellular developmental process, cell cycle DNA replication, divalent metal ion transport, DNA metabolic process, genetic transfer
Cell cycle G2/M	Microtubule-based process, mitotic cytokinetic process, histone lysine methylation, floral organ formation, nuclear division, DNA alkylation, histone modification, regulation of DNA metabolic process, single-organism cellular process, regulation of cell cycle
Palisade mesophyll	Sulfur amino acid biosynthetic process, rRNA metabolic process, generation of precursor metabolites and energy, cellular protein complex assembly, metal ion transport, cellular ion homeostasis, organelle organization, response to red or far-red light, regulation of protein modification process, cellular protein modification process
Spongy mesophyll	Gene expression, pyruvate metabolic process, response to bacterium, response to red or far-red light, cellular protein complex assembly, NADP metabolic process, rRNA metabolic process, RNA modification, generation of precursor metabolites and energy, sulfur amino acid biosynthetic process
Pavement cell	Response to organonitrogen compound, response to acid chemical, response to stress, response to osmotic stress, single-organism metabolic process, response to disaccharide, immune effector process, response to fungus, response to other organism, protein targeting
Guard cell	Response to stress, multicellular organism development, developmental process involved in reproduction, response to acid chemical, fatty acid metabolic process, protein targeting, regulation of programmed cell death, response to osmotic stress, cell morphogenesis involved in differentiation, response to radiation, response to organonitrogen compound
Cambium cell	Response to metal ion, regulation of gene expression, developmental process involved in reproduction, response to osmotic stress, sulfur amino acid biosynthetic process, response to bacterium, response to water deprivation, cellular macromolecule biosynthetic process, single-organism metabolic process, hormone-mediated signaling pathway
Xylem	Regulation of gene expression, developmental process involved in reproduction, cellular macromolecule biosynthetic process, hormone-mediated signaling pathway, meristem structural organization, single-organism metabolic process, external encapsulating structure organization, auxin transport, response to osmotic stress, response to hormone
Phloem	Response to osmotic stress, response to metal ion, developmental process involved in reproduction, response to organonitrogen compound, regulation of gene expression, cellular macromolecule biosynthetic process, sulfur amino acid biosynthetic process, defense response, single-organism metabolic process, hormone-mediated signaling pathway
Companion cell	Response to osmotic stress, response to light intensity, hormone-mediated signaling pathway, response to metal ion, regulation of gene expression, cellular macromolecule biosynthetic process, response to stress, response to water deprivation, indoleacetic acid metabolic process, response to hormone
Sieve element	Primary metabolic process, single-organism metabolic process, organelle organization, hormone-mediated signaling pathway, sulfur amino acid biosynthetic process, polysaccharide catabolic process, response to acid chemical, response to stress, indoleacetic acid metabolic process, response to organonitrogen compound
Bundle sheath	Response to organonitrogen compound, hormone-mediated signaling pathway, response to acid chemical, response to stress, immune effector process, regulation of programmed cell death, protein targeting, regulation of gene expression, response to fungus, response to other organism

**Table 2.** Functional analysis of previously unreported marker genes in 15 leaf populations

Target_Cluster	Gene ID	Gene name	GO function
Cell cycle G1/S	Manes_13G097600	HTR2	GO:0003676//nucleic acid binding; GO:0046983//protein dimerization activity
	Manes_16G016800	At4g27230	GO:0003676//nucleic acid binding; GO:0046983//protein dimerization activity
	Manes_05G070300	HIS2A	GO:0003676//nucleic acid binding; GO:0046983//protein dimerization activity
	Manes_18G018600	HTR2	GO:0003676//nucleic acid binding; GO:0046983//protein dimerization activity
	Manes_01G069400	At1g07660	GO:0003676//nucleic acid binding; GO:0046983//protein dimerization activity
Cell cycle G2/M	Manes_01G147400	At3g51280	-
	Manes_15G015100	Manes_15G015100	-
	Manes_01G096000	Manes_01G096000	-
	Manes_03G008300	At5g25090	GO:0046914//transition metal ion binding
	Manes_09G054700	At3g28850	GO:0015036//disulfide oxidoreductase activity
Palisade mesophyll	Manes_12G041300	ARP1	GO:0097159//organic cyclic compound binding
	Manes_03G155600	Manes_03G155600	-
	Manes_02G109200	PIP2-7	-
	Manes_13G146300	Manes_13G146300	-
	Manes_05G081100	CHLASE1	-
Spongy mesophyll	Manes_16G096200	ABP19A	GO:0016616//oxidoreductase activity, acting on the CH-OH group of donors, NAD or NADP as acceptor; GO:0016623//oxidoreductase activity, acting on the aldehyde or oxo group of donors, oxygen as acceptor; GO:0046914//transition metal ion binding; GO:0048037//cofactor binding
	Manes_01G174400	Manes_01G174400	-
	Manes_05G130500	CAT2	GO:0004601//peroxidase activity; GO:0046906//tetrapyrrole binding; GO:0046914//transition metal ion binding; GO:0046943//carboxylic acid transmembrane transporter activity
	Manes_16G123200	At4g00165	GO:0005488//binding; GO:0016787//hydrolase activity
	Manes_15G067700	VTC2	GO:0004645//phosphorylase activity; GO:0008905//mannose-phosphate guanylyltransferase activity; GO:0035251//UDP-glucosyltransferase activity
Pavement cell	Manes_01G112700	LTP-2	GO:0005488//binding
	Manes_15G016300	RCOM_0680180	-
	Manes_06G165200	ALDH3F1	GO:0016620//oxidoreductase activity, acting on the aldehyde or oxo group of donors, NAD or NADP as acceptor
	Manes_17G062300	At1g29670	GO:0016788//hydrolase activity, acting on ester bonds
	Manes_05G090000	MLP423	-
Guard cell	Manes_16G117500	Manes_16G117500	-
	Manes_02G037500	TPRP-F1	GO:0005488//binding; GO:0016787//hydrolase activity
	Manes_11G069500	WAKL8	-
	Manes_04G015900	Manes_04G015900	-
	Manes_06G024400	CER3	GO:0003824//catalytic activity; GO:0046914//transition metal ion binding
Cambium cell	Manes_03G099400	Manes_03G099400	-
	Manes_08G030200	Os04g0179200	GO:0033764//steroid dehydrogenase activity, acting on the CH-OH group of donors, NAD or NADP as acceptor
	Manes_02G018200	SP1L5	-
	Manes_03G099100	Manes_03G099100	-
	Manes_18G043700	SN1	-
Xylem	Manes_14G066900	PGL3	-
	Manes_01G144500	NAC037	GO:0001071//nucleic acid binding transcription factor activity
	Manes_14G164200	Manes_14G164200	-
	Manes_14G097100	BHLH30	GO:0005515//protein binding
	Manes_05G132600	Manes_05G132600	GO:0005488//binding
Phloem	Manes_04G116400	Manes_04G116400	GO:0016772//transferase activity, transferring phosphorus-containing groups; GO:0046914//transition metal ion binding
	Manes_14G107900	Manes_14G107900	-
	Manes_18G068600	Manes_18G068600	-
	Manes_13G110700	Manes_13G110700	-
	Manes_02G200000	Os02g0778400	GO:0016776//phosphotransferase activity, phosphate group as acceptor; GO:0019201//nucleotide kinase activity; GO:0032550//purine ribonucleoside binding
Companion cell	Manes_12G133000	UGT85K5	GO:0035251//UDP-glucosyltransferase activity
	Manes_17G112400	Manes_17G112400	-
	Manes_12G132900	CYP71E7	-
	Manes_01G202400	DJ1E	GO:0016787//hydrolase activity
	Manes_09G108300	Manes_09G108300	-
Sieve element	Manes_03G128000	At2g25060	GO:0046914//transition metal ion binding
	Manes_14G053600	BGLU24	GO:0015926//glucosidase activity
	Manes_08G154500	Manes_08G154500	-
	Manes_12G136500	PLAT1	-
	Manes_10G083400	MYC4	-
Bundle sheath	Manes_01G113100	Manes_01G113100	-
	Manes_01G220400	ARG2	-
	Manes_01G230000	WRKY23	GO:0003677//DNA binding
	Manes_01G121300	PUB21	GO:0003824//catalytic activity
	Manes_17G051600	PTI5	GO:0001071//nucleic acid binding transcription factor activity; GO:0003676//nucleic acid binding

### Differentiation trajectories of cassava leaves

**Figure 6.**
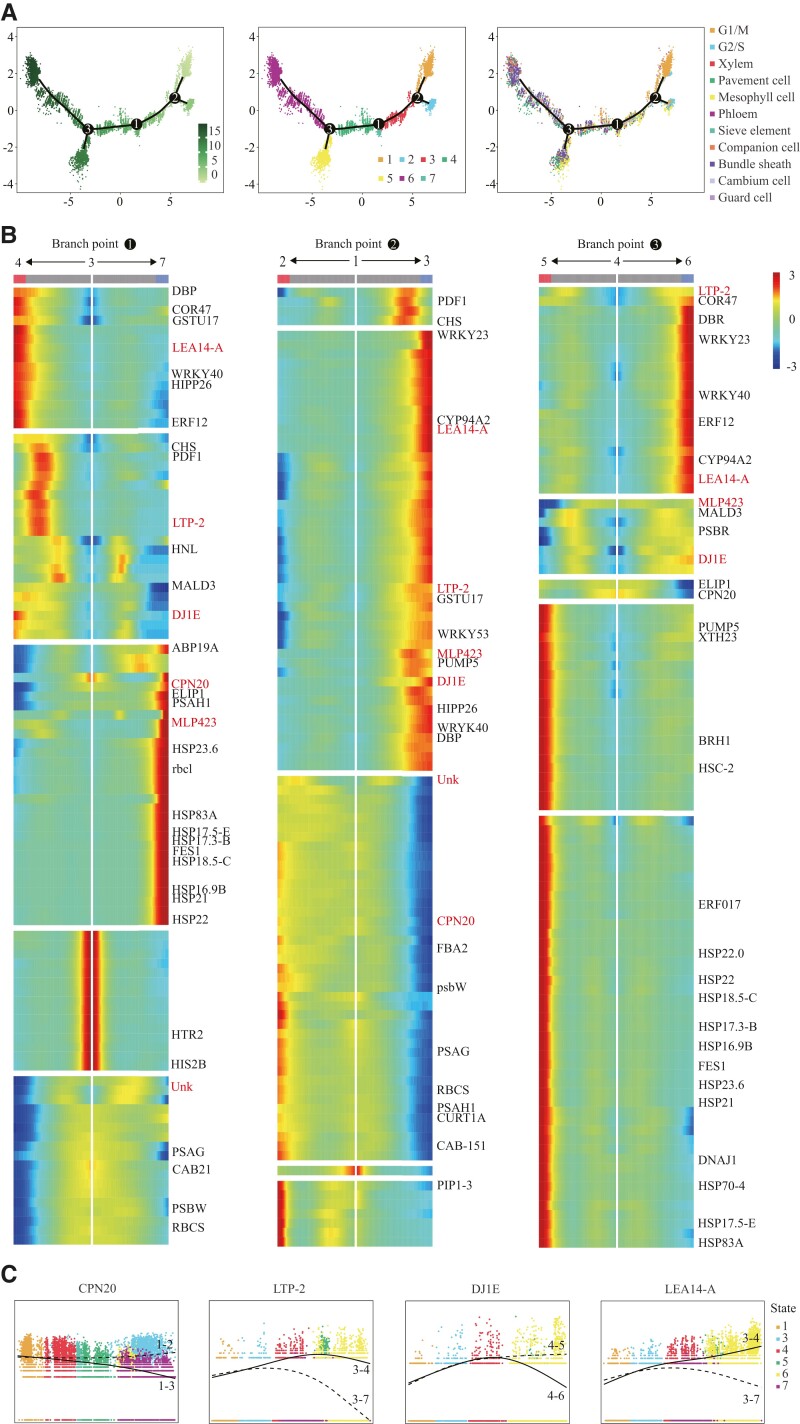
Pseudotime analysis of differentiation trajectory and cell fate within cell-type populations. **A)** The cell ordering along the differentiation trajectory successively presented by pseudotime states, branch states, and cell types. **B)** Heatmap of the top 100 substantially changed genes discovered by the BEAM function from monocle in 3 branch points. Detailed information of these genes is given in Supplemental Table S16. **C)** Representative genes of 3 branch points were selected to show their expression trends before and after cell differentiation.

To verify cell types and explore the continuous differentiation trajectory of cassava leaves, we used all types of cell populations for pseudotime analysis. Pseudotime analysis showed that the pseudotime trajectory had 3 branch points, and all cells were divided into 7 branches (Fig. 6A). The G1/S phase cells were located at the starting point of the pseudotime differentiation trajectory (Fig. 6A). Epidermal cells were distributed in 6 branches except Branch 1, and guard cells are distributed in Branch 6 (Fig. 6A). The mesophyll cells mainly occupied Branches 2, 3, 4, 5, and 7 (Fig. 6A). All vascular system cells were distributed in Branch 6 (Fig. 6A). We further analyzed the key genes related to the growth and development of cassava leaves. Among the top 50 genes were related to ribosome composition (Supplemental Table S15). Branched expression analysis modeling (BEAM) results provide us with a list of genes with substantial changes, which may determine or reflect the fate of cells before and after the 3 branching points (Supplemental Table S16). Six genes appeared during the 3 branch points, including genes involved in lipid transfer and deposition, oxidative stress response, abscisic acid (ABA) signal transduction, and 1 gene with unknown function (Fig. 6, B and C). The thermal map of genes with significant changes was detected at 3 branch points (*P* < 0.01), which showed the gene expression pattern when cell fate was determined. In the above results, we mapped the cell differentiation and development trajectory of cassava leaves and annotated the high-expression genes of each state and branching point. In branch point 2, from Branch 1 to Branch 2, the highly expressed genes were mainly ribosome and photosynthesis-antenna protein-related genes; from Branch 1 to Branch 3, the highly expressed genes include genes involved in glutathione metabolism, pyruvate metabolism, α-linolenic acid metabolism, cutin, and wax biosynthesis (Fig. 6B). In branch point 1 and branch point 3, heat shock protein family genes were highly expressed in States 7 and 5, indicating that heat shock proteins played an important role in plant growth and development (Fig. 6B). Vascular system cells differentiated specifically in State 6. The genes highly expressed in this stage include genes involved in material transport, such as *Polyubiquitin* (*UBQ3*), *ferritin-4* (*PFE2*), *21 kDa protein* (*PMEI11*), *probable aquaporin PIP-type 7a* (*TRG-31*), and *ethylene-responsive transcription factor 12* (*ERF12*) (Fig. 6B). Branches with epidermal cells highly express genes associated with stress and stimulation (Fig. 6B). These results indicated that leaf cells differentiate into different types of cells by expressing different functional genes.

### Cell specificity of genes related to secondary metabolites in cassava leaves during leaf development

**Figure 7.**
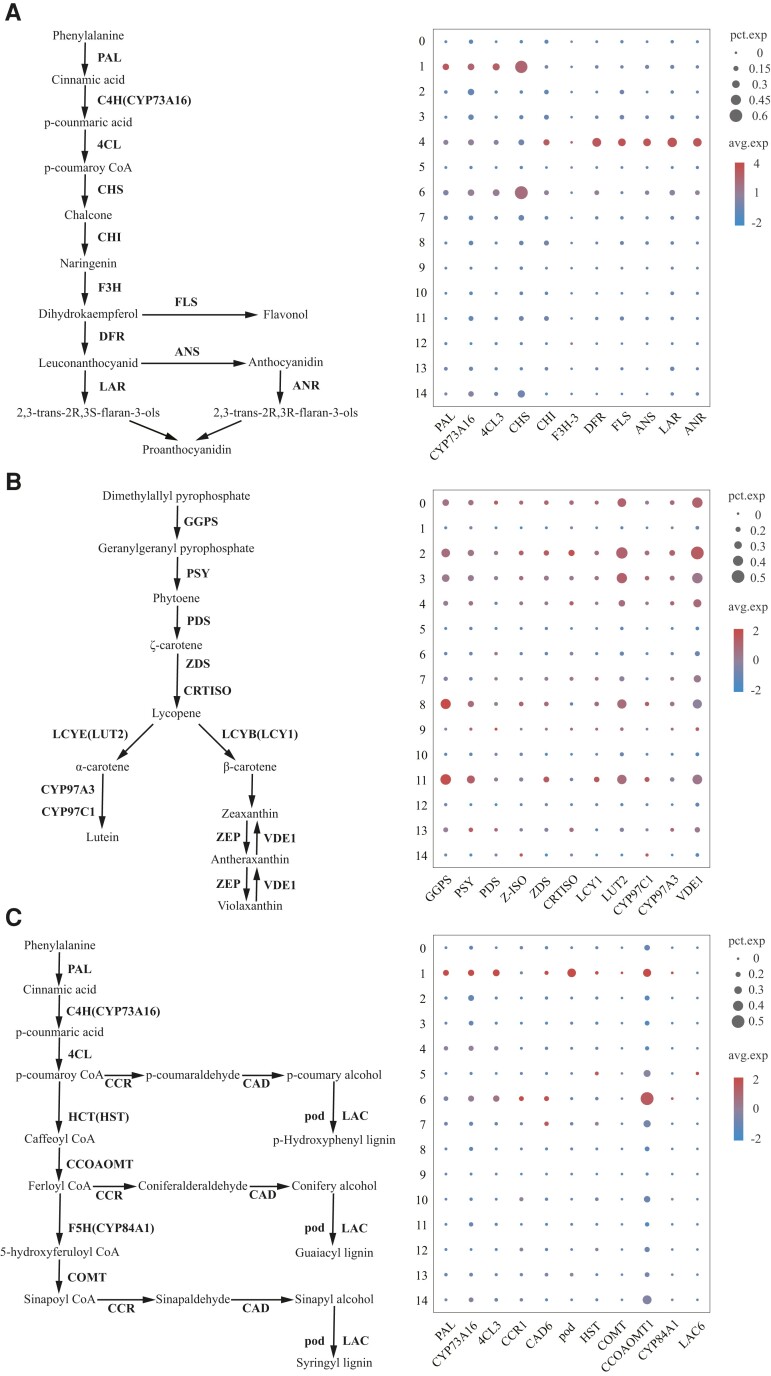
Cell-specific distribution of genes related to the biosynthesis of flavonoids, proanthocyanidins, carotenoids, and lignin. **A)** Schematic diagram of the flavonoids and proanthocyanidin biosynthesis pathway. Dotplot with flavonoids and proanthocyanidin biosynthesis genes of each cell cluster. **B)** Schematic diagram of the carotenoid biosynthesis pathway. Dotplot with carotenoid biosynthesis genes of each cell cluster. **C)** Schematic diagram of the lignin biosynthesis pathway. Dotplot with lignin biosynthesis genes of each cell cluster.

Secondary metabolites in plants play an important role in plant growth and stress resistance. The secondary metabolites in leaves include flavonoids, proanthocyanidins, carotenoids, and lignin. We visualized the expression of genes involved in these secondary metabolic biosynthetic pathways (Supplemental Table S17). We found that genes involved in flavonoid, proanthocyanidin, and carotenoid biosynthesis were highly expressed in mesophyll cells (Fig. 7, A and B). The flavonoid and proanthocyanidin biosynthetic genes, *flavanone 3-dioxygenase 3* (*F3H-3*), *lavonol synthase/flavanone 3-hydroxylase* (*FLS*), *leucoanthocyanidin dioxygenase* (*ANS*), and *anthocyanidin reductase* (*ANR*), were only highly expressed in sponge mesophyll cells (Fig. 7A). Upstream genes involved in lignin and flavonoid biosynthesis are *phenylalanine ammonia-lyase* (*PAL*) and *4-coumarate-CoA ligase 2* (*4CL3*), both of which were highly expressed in epidermal cells (Fig. 7, A and C). The downstream genes of lignin biosynthesis were highly expressed in the epidermis and vascular cells. *Cinnamoyl-CoA reductase 1* (*CCR1*), *cinnamyl alcohol dehydrogenase 1* (*CAD1*), *cinnamyl alcohol dehydrogenase 9* (*CAD9*), and *peroxidase 15* (*pod*) were highly expressed in epidermal cells (Fig. 7C). *Laccase-17* (*LAC17*), *laccase-6* (*LAC6*), *laccase-14* (*LAC14*), and *caffeic acid 3-O-methyltransferase* (*COMT*) were highly expressed in vascular cells (Fig. 7C). *Shikimate O-hydroxycinnamoyltransferase* (*HST*), *caffeoyl-CoA O-methyltransferase* (*CCOAOMT1*), and *cytochrome P450 84A1* (*CYP84A1*) were highly expressed in epidermal and vascular cells (Fig. 7C). Lignin can enhance the mechanical strength of tissues, and the specific expression of these genes was consistent with the characteristics of epidermal and vascular cells. Our results showed that the expression of genes involved in plant secondary metabolite biosynthesis is specific in different types of cells.

## Discussion

Due to the hard cell walls of plant cells, it is difficult to isolate protoplasts (Efroni and Birnbaum 2016). Therefore, the current plant scRNA-seq research mainly focuses on some tissues that are relatively easy to isolate protoplasts from Chen, Lv, et al. (2021) and Chen, Tong, et al. (2021). For example, most cell types were identified in *Arabidopsis* and rice roots, determining the expression characteristics of root cells during differentiation (Zhang et al. 2019; Liu, Hu, et al. 2021; Liu, Liang, et al. 2021). In addition to the study of model plant leaves, protoplasts have been successfully isolated from nonmodel plant leaves and their single-cell transcriptional maps have been constructed (Kim et al. 2021; Liu, Hu, et al. 2021; Liu, Liang, et al. 2021; Wang et al. 2021). This study constructs a single-cell transcriptome atlas of cassava leaves. Through the analysis of single-cell transcriptome data, 15 unique cell populations were identified. In this study, trichome cells were not identified, probably because the trichome cell wall with lignin and pectin is difficult to avoid mRNA degradation in isolation (Marks et al. 2008; Becker et al. 2022). In addition, only a few cassavas were found trichome by microscopic observation of leaves of different cassava varieties (Marin et al. 2020), and SC8 cassavas had microscopically observed trichomes (Supplemental Fig. S1), so there was no single-cell transcriptome data on trichomes. The results of this study provide an important theoretical basis for the identification and classification of leaf tissue cells of shrubs and starch crops.

In the scRNA-seq study of *Arabidopsis* and rice, cell group annotations are obtained from gene expression data that have biological functions or expression patterns that have been fully studied (Zhang et al. 2021). Since cassava has no related cell marker genes, we manually annotate each tissue cell according to the marker genes in other plants. The identification of cassava leaves based on multiple marker genes will be very important in future studies, especially if scRNA-seq analysis is performed under stress or using genetic perturbations, as it may affect the expression of some classical marker genes. In order to make it easier for clustering annotation in future studies on cassava scRNA-seq, we identified additional marker genes for each tissue. Mesophyll cells are mainly defined by the expression of photosynthesis-related genes, but abiotic stresses such as oxidative stress substantially affect leaf photosynthetic efficiency (Nouri et al. 2015). In this case, marker genes unrelated to photosynthesis may contribute to tissue identification. We found that *auxin-binding protein ABP19a* (*ABP19A*) can be used as a candidate marker gene for mesophyll cells (Fig. 2). ABP19A is an important regulator of the auxin signaling pathway, which can regulate the response to auxin and play an important role in plant growth and development (Peinado-Guevara et al. 2017). We found *aldehyde dehydrogenase family 3 member F1* (*ALDH3F1*) in epidermal cells. Studies have found that there is no substantial difference in the expression of ALDH3F1 in osmotic stress (Kirch et al. 2005). It indicates that ALDH3F1 has the potential to be a marker gene for epidermal cells. These results provide a basis for cell identification of cassava tissue.

In the leaves of C3 plants, chloroplasts are mainly concentrated in mesophyll cells, so photosynthesis of C3 plants only occurs in mesophyll cells, while BS cells in the leaves of C4 plants contain more chloroplasts, so BS cells become the main site for photosynthesis of C4 plants (Cui 2021). Compared with C3 plants, C4 plants have cell-specific photosynthesis-related genes, *phosphoenolpyruvate carboxylase* (*PEPC*), *malate dehydrogenase* (*NADP-MDH*), and *carbonic anhydrase* (*CA*) are specifically expressed in mesophyll cells, while *RBCS*, *dicarboxylate transporter 1* (*DIT1*), and *NADP-dependent malic enzyme* (*NADP-ME*) are highly expressed in BS cells (Cui 2021; Taniguchi et al. 2021). In our study, NADP-MDH, CA, RBCS, and DIT1 were specifically expressed in mesophyll cells, while PEPC and NADP-ME were highly expressed in vascular tissues. We found that the photosynthesis of sc8 cassava leaves mainly occurs in mesophyll cells, which is consistent with the characteristics of C3 plants, but some photosynthesis-related genes are also expressed in vascular cells, which also indicates that SC8 cassava leaves have some C4 plant characteristics. These results indicate that SC8 cassava is a cassava variety between C3 and C4 plants, which can provide important insights into the evolution of C3 plants to C4 plants.

The construction of the leaf cell differentiation trajectory by scRNA-seq is conducive to a better description of leaf cell development. The development of plant leaves to the final shape requires cell proliferation, cell expansion, and cell differentiation, and the final size of the leaves depends on the balance between cell proliferation and cell expansion (Dewitte et al. 2003; Blomme et al. 2014). Cell proliferation generally occurs in meristem regions, characterized by vigorous cell division and the potential to develop into different cells (Inze and De Veylder 2006). After the leaves develop to a specific time, the cells stop proliferating and begin to expand to promote further growth of the leaves (Vercruysse et al. 2020). During plant development, many differentiated cells undergo multiple DNA replications without cell division, known as the endoreplication stage (Breuer et al. 2010, 2014). The process of DNA replication in endoreplication is the same as that in the cell cycle (De Veylder et al. 2011). In this study, the G1/S phase cells are located at the beginning of the pseudotime trajectory. This cell population is a cell that is undergoing DNA replication, but no meristematic cell marker gene can identify this subgroup. Therefore, it is inferred that this cell population may contain cells in the cell cycle and cells in the inner cycle. Interestingly, we also found that both G1/S and G2/M cells have mesophyll cell characteristics. Studies have found that chloroplasts can affect cell proliferation and cell expansion, which is one of the key factors regulating cell proliferation and cell expansion (Andriankaja et al. 2012). Moreover, the chloroplast is also the site of photosynthesis, which may provide efficient energy for cell cycle progression (Hudik et al. 2014). Therefore, we speculate that the cells in the early stage of development may be proliferative cells with chloroplasts. During development, chloroplasts play different roles according to the different functions of differentiated cells. In the epidermal cell population, chloroplasts can be seen in the early development of epidermal cells, while mature epidermal cells lose photosynthetic activity after differentiation (Charuvi et al. 2012; Barton et al. 2018). This phenomenon was also found in our study. Our results can provide a theoretical basis for further understanding of plant leaf development.

scRNA-seq can be used not only for the basic research of cell heterogeneity but also for the analysis of crop germplasm resources for improvement. In particular, through the analysis of key gene regulatory factors for crop growth and development, the improvement of crop germplasm varieties has been greatly improved (Nadolska-Orczyk et al. 2017). In addition to revealing cell-heterogeneous gene expression atlas, scRNA-seq will be used in many areas of plant research, such as plant responses to biotic and abiotic stresses. Transcriptome changes caused by environmental stress are widely detected in plant tissues, but some stress-induced transcriptome changes have not been explored at the single-cell level. By combining single-cell isolation and transcriptome analysis, the changes in transcription expression levels in single cells can be detected to better understand the application of plants to environmental stress (Mo and Jiao 2022). Leaves are the most important aboveground tissue of plants, which is vulnerable to the invasion of plant pathogens (Weerawanich et al. 2018). Further integration of scRNA-seq with the overall RNA-seq of plant leaves will help identify the main cell groups involved in immune resistance pathways. Although the application of scRNA-seq is still limited by the isolation method of single plant cells, the rapid progress in this aspect will reveal the plant development mechanism that has not been explored so far.

## Materials and methods

### Isolation of plant materials and protoplasts

In this study, the cassava (*M. esculenta* Crantz) variety “SC8” was selected by the Institute of Biology at Hainan University. The third leaf (6.8 ± 0.2 cm in length and 9.0 ± 0.3 cm in width) of the cassava stem tip was cut into fragments of about 1 mm. A 5-mL enzymolysis solution (0.02 M KCl, 0.01 M CaCl_2_, 0.025-g bovine serum albumin, 0.01 M MES, 0.5 M mannitol solution, 0.08-g cellulase, 0.005-g pectinase, 0.5-mL PVP-4, 0.05-g macerozyme, and 0.05-g snailase) was added to the fragments, and vacuum suction was performed on a vacuum device for 5 min. The mixture was incubated in an incubator at 20 °C and 200 × *g* for 2 h to release protoplasts from cassava leaves. The protoplasts were filtered through a 70-*µ*m cell sieve, centrifuged at 100 × *g* for 2 min, and washed twice with 0.4 M mannitol. After removing the supernatant, a small amount of 0.4 M mannitol solution was added to suspend the protoplast. The cells were further filtered by a 40-*µ*m cell sieve, and the appropriate amount of protoplast suspension was mixed with trypan blue solution at a ratio of 9:1. Then, the Countess II Automated Cell Counter was used to count cells and calculate the proportion of live cells, while ensuring the cell viability was 90% and the cell density around 800 to 900 cells/*µ*L.

### scRNA-seq library construction and sequencing

Gel beads containing barcode information were combined with a mixture of cells and enzymes. Cellular suspensions were loaded on a 10X Genomics GemCode single-cell instrument and then wrapped in oil surfactant droplets located in a microfluidic “double-cross” system to form GEMs (Gel Beads-In-Emulsions). GEMs flowing into the reservoir were collected, then lysed to release barcode sequences, reverse transcribed to obtain cDNA fragments, and labeled. Full-length, barcoded cDNAs were then amplified by PCR to generate sufficient mass for library construction. The indexed sequencing libraries were prepared using Chromium Single Cell 3′ Reagent Kits (v2) according to the manufacturer's instructions. Finally, library sequencing was performed by Illumina HiSeq 4000.

### Data processes

We used 10X Genomics Cell Ranger software (version 3.1.0) to compare and quantify the data. The low-quality bar code and UMI reading filter were used to map the reference genome of Cassava (JGI-v6.1). The cell-by-gene matrices for each sample were individually imported to Seurat version 3.1.1 for downstream analysis. Cells with an unusually high number of UMIs (≥8,000) or mitochondrial gene percent (≥10%) were filtered out. After deleting unwanted cells from the data set, we used the “LogNormalize” global scaling normalization method to normalize the gene expression measurement of each cell and then logarithmically converted the results. The data were used for UMAP and t-SNE to cluster in a 2D space. Cells with similar expression patterns were clustered based on a graph-based clustering method. We used the likelihood-ratio test to find differential expression for a single cluster compared to all other cells. We identified DEGs by the following criteria: (i) *P* ≤ 0.01; (ii) log_2_ (fold change [FC]) ≥ 0.360674; and (iii) the percentage of cells where the gene is detected in a specific cluster >25%. Further GO enrichment analysis and Kyoto encyclopedia of genes and genomes (KEGG) pathway enrichment analysis were performed based on gene expression levels to determine the primary functions of these clusters.

### Marker gene and cell-type identification

In order to determine the cell types of cassava leaves, cell populations were identified by cell-specific marker genes. We aligned the cell type marker genes reported in *Arabidopsis* (*A. thaliana*) to the corresponding genes of cassava by homologous gene alignment. The types of cell populations were determined and annotated by using the expression changes of these marker genes in each cell population. The identified cell types were used for subsequent differential gene analysis of different cell populations.

### Pseudotime analysis

In order to understand the changes of genes during the development of cassava leaves, the single-cell pseudotime differentiation trajectory of cassava leaves was constructed by Monocle (Version 2.0). We used the expression matrix of all cell populations for pseudotime analysis. The genes with large differences or variations were retained, and the cell differentiation trajectories were constructed in the dimensionality reduction space starting from the G1/S phase cell population. We further analyzed the gene expression of branch changes by BEAM. Subsequently, we analyzed key genes related to development and differentiation processes and performed cluster analysis and KEGG/GO annotation.

### In situ hybridization

In this study, gene-specific probes were first prepared according to the Dig Northern Starter Kit manual (Roche), and the primers used in this study are listed in Supplemental Table S18. The leaves, with the same growth period, were fixed in 50% Formaldehyde-acetic acid-ethanol fixative (FAA) at 4°C for 24 h, after which the samples were embedded. Paraffin-embedded samples were sliced with a sliding slicer (Leica) with a thickness of 10 microns. The sections were placed in Xylene I for 15 min, Xylene II for 15 min, anhydrous ethanol for 10 min, and diethyl pyrocarbonate (DEPC) water for 5 min. The slices were boiled in the repair solution for 10 min, cooled naturally, and digested with proteinase K (20 *µ*g/mL) at 37 °C for 10 min. After washing with phosphate buffer solution (PBS) for 15 min, the membrane-breaking solution was added to the tissue for 20 min. Salmon sperm liquid was added to the hybridization solution at a concentration of 1/100 and then added to the slice at 37 °C for 1 h. After removing the prehybridization solution, the hybridization solution containing the probe was allowed to hybridize to the slice at 42 °C overnight. We washed the hybridization solution off and analyzed the slice under the microscope (NIKON Eclipse 80i).

### Accession numbers

Sequence data from this article can be found in the GenBank/EMBL data libraries under accession numbers: *LHCB4.1* (CM004394), *LHCB5* (CM004401), *RBCS* (CM004391), *PSAE1* (CM004398), *PSBP* (CM004388), *KCS1* (CM004391), *FDH* (CM004402), *KCS20* (CM004401), *GDU4* (CM004388), *YAB5* (CM004392), *CHS* (CM004397), *FAMA* (CM004389), *MYB60* (CM004395), *YAB1* (CM004387), *RBR* (CM004402), *TIR1* (CM004391), *AFB2* (CM004397), *H2B-3* (CM004393), *PCNA* (CM004387), *CDKB1-2* (CM004394), *CDKB2-2* (CM004391), *NAD-ME2* (CM004387), *PPCK1* (CM004401), *DOF5.6* (CM004392), *SEOB* (CM004403), *SUC2* (CM004404), *NAKR2* (CM004397), *ACL5* (CM004402), *PIN6* (CM004404), *CESA8* (CM004402), *COBL4* (CM004392), *WOX4* (CM004404), *TDR* (CM004400), *Protein SCR* (CM004394), *SCL23* (CM004394), *UBQ3* (CM004403), *PFE2* (CM004394), *PMEI11* (CM004400), *TRG-31* (CM004393), *ERF12* (CM004403), *F3H-3* (CM004400), *FLS* (CM004404), *ANS* (CM004387), *ANR* (CM004402), *PAL* (CM004394), *4CL3* (CM004395), *CCR1* (CM004396), *CAD1* (CM004400), *CAD9* (CM004398), *pod* (CM004401), *LAC17* (CM004390), *LAC6* (CM004391), *LAC14* (CM004404), *COMT* (CM004398), *HST* (CM004390), *CCOAOMT1* (CM004396), *CYP84A1* (CM004387), *ABP19A* (CM004397), *ALDH3F1* (CM004392), *PEPC* (CM004388), *NADP-MDH* (CM004393), *CA* (CM004401), *DIT1* (CM004392), and *NADP-ME* (CM004390).

## Supplementary Material

kiad500_Supplementary_Data

## Data Availability

The single cell transcriptome dataset of the Cassava leaves that was used in the article is available at the Genome Sequence Archive (GSA) database of National Genomics Data Center (NGDC) (https://ngdc.cncb.ac.cn/). The accession number for the dataset is CRA012723.

## Supplemental data

The following materials are available in the online version of this article.

**Supplemental Figure S1.** Brief chart of Cell Ranger software report.

**Supplemental Figure S2.** Data quality control determined the medium number of genes and UMI.

**Supplemental Figure S3.** t-SNE representation of the cassava leaf transcriptomic landscape profiled by scRNA-seq.

**Supplemental Figure S4.** GO function enrichment analysis of Cluster #1 and Cluster #6.

**Supplemental Figure S5.** Photomicrographs of leaf transverse sections of SC8 cassava.

**Supplemental Figure S6.** Bright-field microscopy observation of cassava leaf sections.

**Supplemental Figure S7.** Expression pattern of additional epidermal cell population markers.

**Supplemental Figure S8.** Expression pattern of additional cell cycle markers.

**Supplemental Figure S9.** Expression pattern of additional vascular cell population markers.

**Supplemental Table S1.** Statistical data of scRNA-seq.

**Supplemental Table S2.** Statistical information of 15 cell clusters.

**Supplemental Table S3.** Reported marker gene for cell clusters.

**Supplemental Table S4.** All identified DEGs in different cell clusters.

**Supplemental Table S5.** Photosynthesis-related genes in different cell clusters.

**Supplemental Table S6.** All identified DEGs in epidermal cell population.

**Supplemental Table S7.** All identified DEGs in mesophyll cell populations.

**Supplemental Table S8.** Auxin-related genes in different cell clusters.

**Supplemental Table S9.** Ethylene-related genes in different cell clusters.

**Supplemental Table S10.** Cell cycle genes in different cell clusters.

**Supplemental Table S11.** Substance transport genes in different cell clusters.

**Supplemental Table S12.** All identified DEGs in vascular cell populations.

**Supplemental Table S13.** Previously unreported marker genes in different cell-type populations.

**Supplemental Table S14.** Top 60 previously unreported marker genes in different cell-type populations.

**Supplemental Table S15.** Top 50 genes of pseudotime analysis.

**Supplemental Table S16.** The top 100 significantly changed genes discovered by the BEAM of 3 differentiation trajectory branch points.

**Supplemental Table S17.** Original data of heatmap for cassava leaves secondary metabolites.

**Supplemental Table S18.** In situ hybridization probe sequence.

## Dive Curated Terms

The following phenotypic, genotypic, and functional terms are of significance to the work described in this paper:

4CL3 Gramene: AT1G65060
4CL3 Araport: AT1G65060

ALDH3F1 Gramene: AT4G36250
ALDH3F1 Araport: AT4G36250

gl3 Gramene: AT5G41315
gl3 Araport: AT5G41315

LHCA5 Gramene: AT1G45474
LHCA5 Araport: AT1G45474

vascular tissue AmiGo: PO:0009015

epidermal cell AmiGo: PO:0004013

mesophyll cell AmiGo: PO:0004006

GL2 Gramene: AT1G79840
GL2 Araport: AT1G79840

DAPI CHEBI: CHEBI:51231

nadp-mdh Gramene: AT5G58330
nadp-mdh Araport: AT5G58330

NADP-ME Gramene: E9NQE2
NADP-ME Araport: E9NQE2
