# Supplementary material for: Single-cell RNA-sequencing profiles reveal the developmental landscape of the Manihot esculenta Crantz leaves
Source: Plant Physiol. 2023 Sep 14;194(1):456–74. doi: 10.1093/plphys/kiad500 (PMC10756766; doi:10.1093/plphys/kiad500)
Supplement: kiad500_Supplementary_Data [file kiad500_supplementary_data.zip › Supplemental figure.pdf]

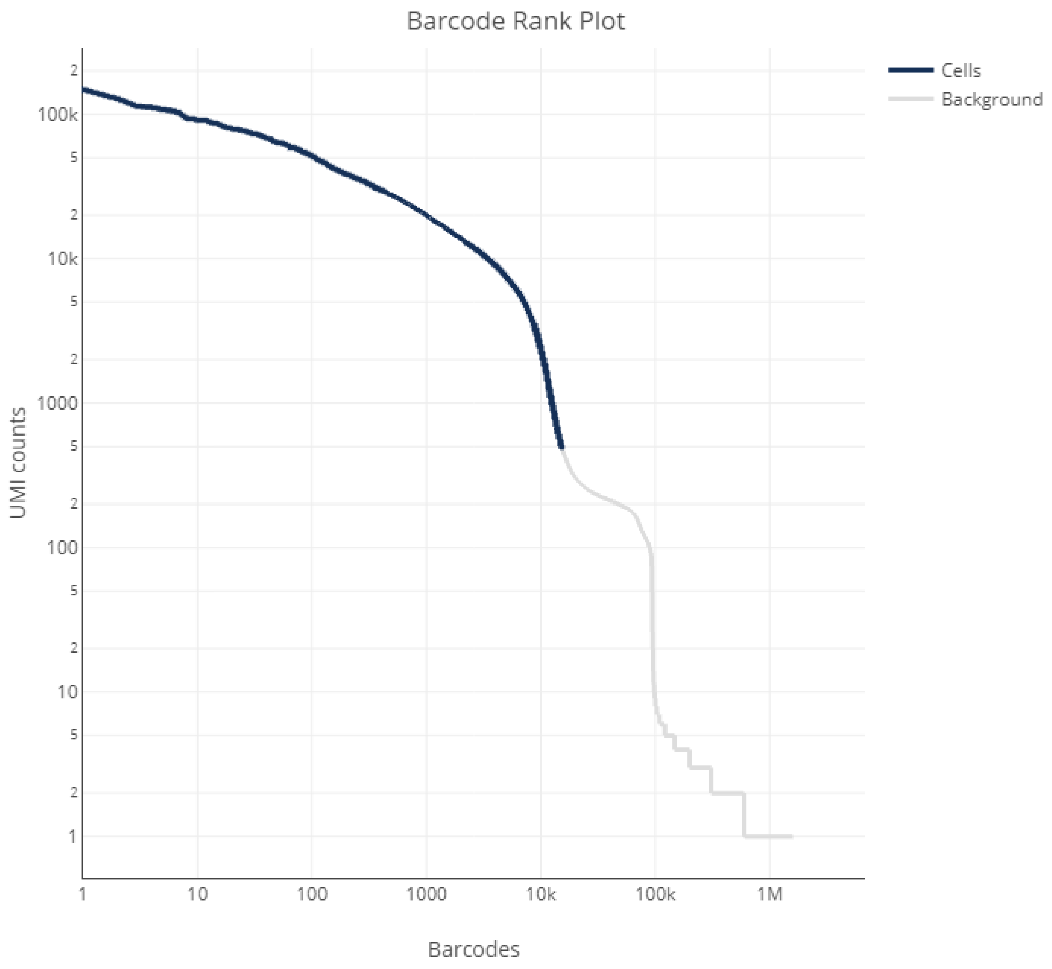

Supplemental Figure S1. Brief chart of Cell Ranger software report.

The abscissa is the number of barcode sequences, and the ordinate is the number of UMI. The barcode corresponding to the green line is the valid cell, and the gray line is the background noise.

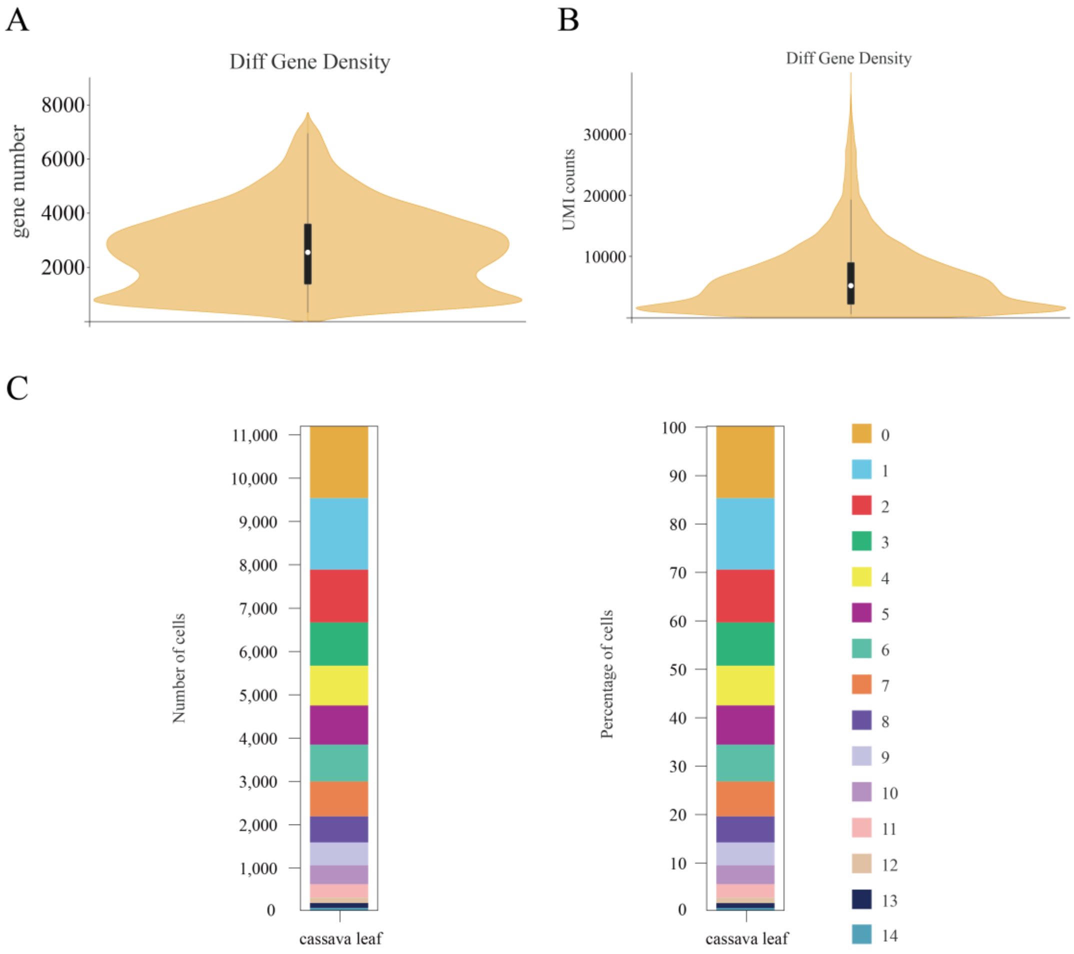

Supplemental Figure S2. Data quality control determined the medium number of gene and UMI. A, The number of genes in cassava leaf cells. B, The number of UMIs in cassava leaf cells. C, The number and percentage of cells in each cassava leaf cell clusters. Different colors represent different cell clusters.

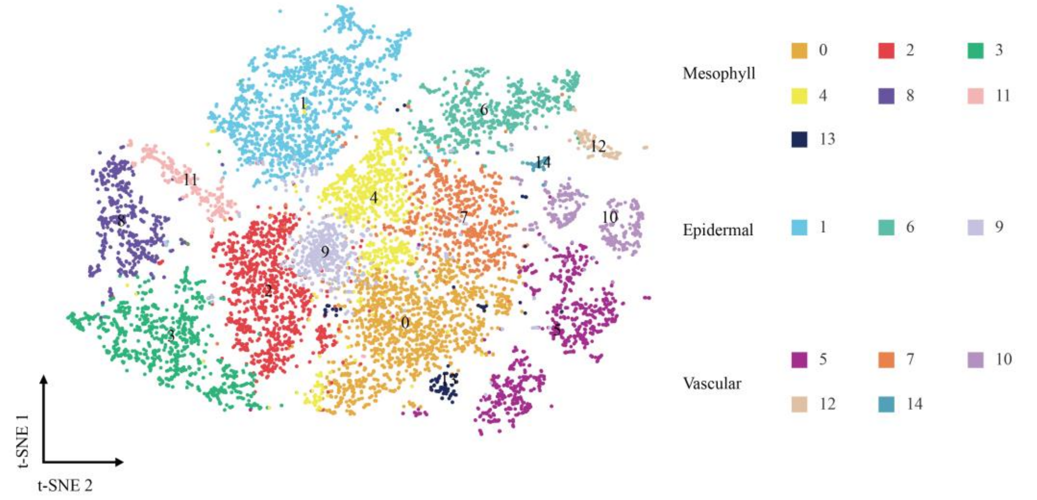

Supplemental Figure S3. t-SNE representation of the cassava leaf transcriptomic landscape profiled by scRNA-sequencing.

Different colors represent different cell clusters.

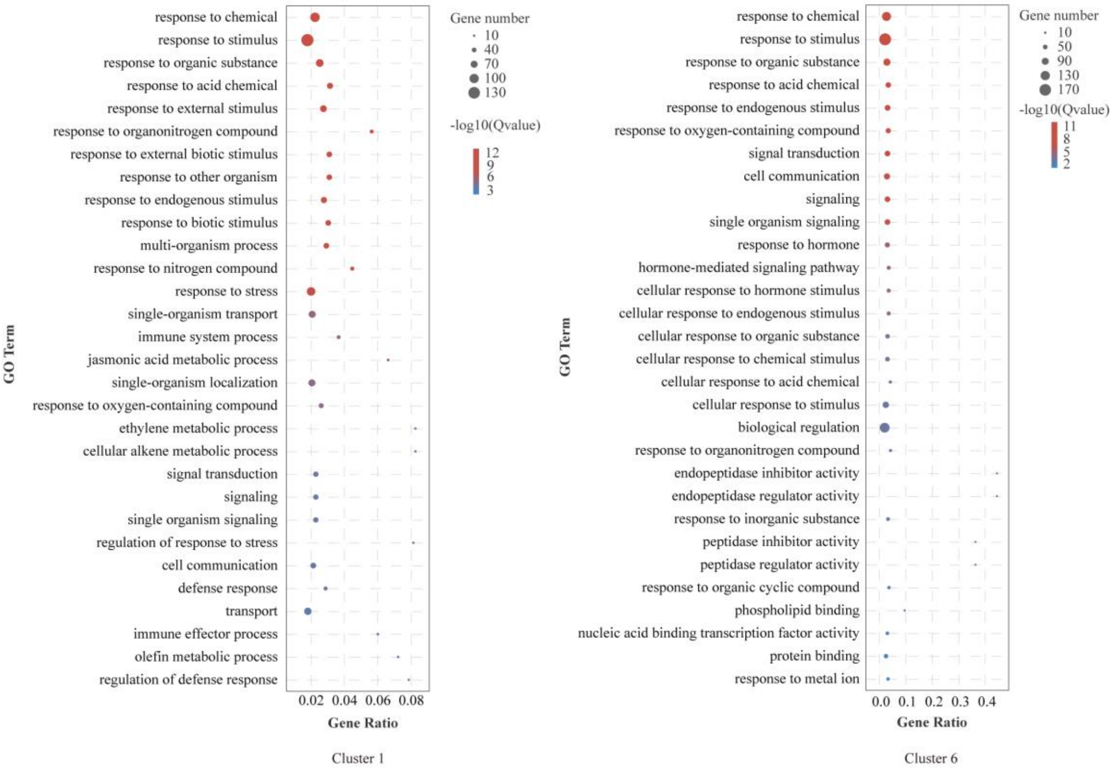

Supplemental Figure S4. GO function enrichment analysis of cluster #1 and cluster #6.

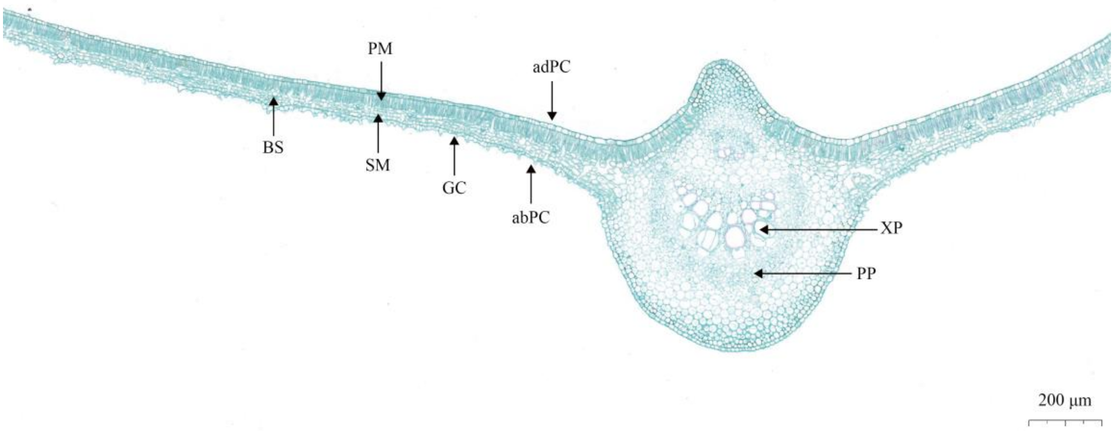

Supplemental Figure S5. Photomicrographs of leaf transverse sections of SC8 cassava.
PM: palisade mesophyll cells; SM: spongy mesophyll cells; adPC: adaxial pavement cells; abPC: abaxial pavement cells; XP: xylem parenchyma; PP: phloem parenchyma; BS: bundle sheaths; GC: guard cells.

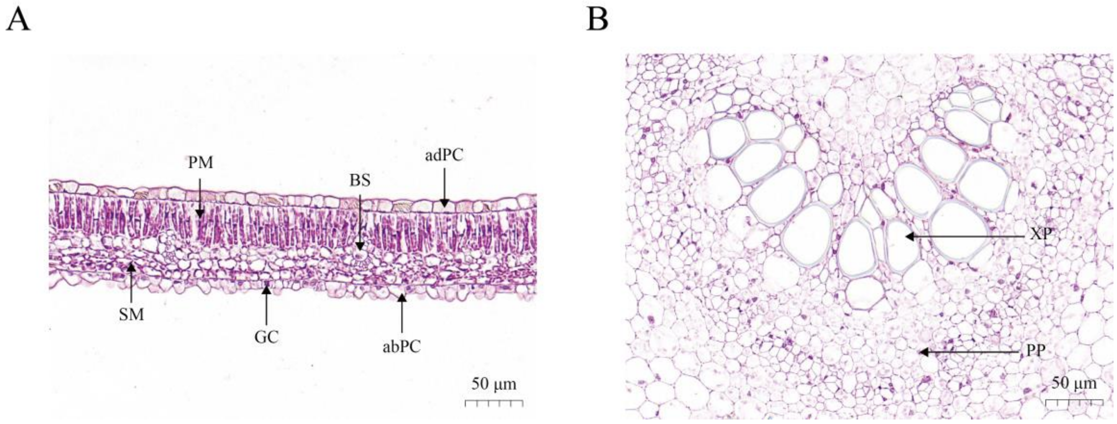

Supplemental Figure S6. Bright-field microscopy observation of cassava leaf sections.

PM: palisade mesophyll cells; SM: spongy mesophyll cells; adPC: adaxial pavement cells; abPC: abaxial pavement cells; XP: xylem parenchyma; PP: phloem parenchyma; BS: bundle sheaths; GC: guard cells.

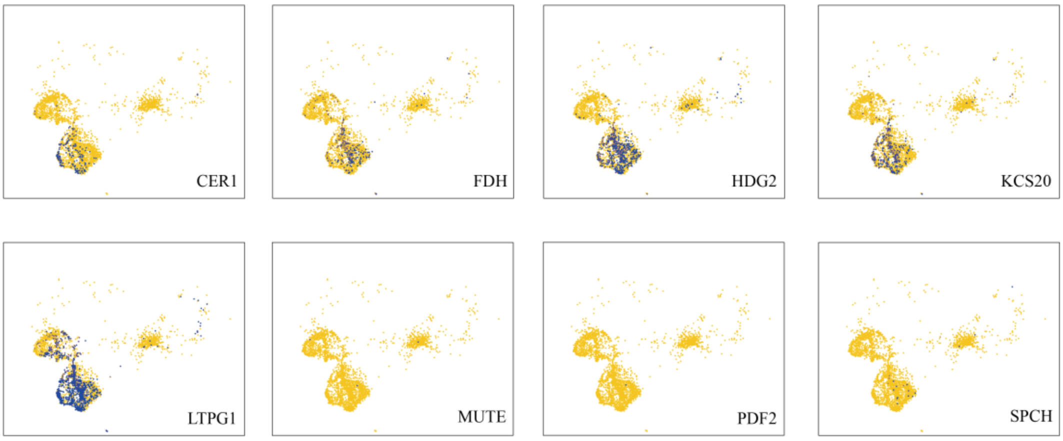

Supplemental Figure S7. Expression pattern of additional epidermal cell populations markers. UMAP plot with the normalized expression of the epidermal cell marker gene (blue = high, yellow = low).

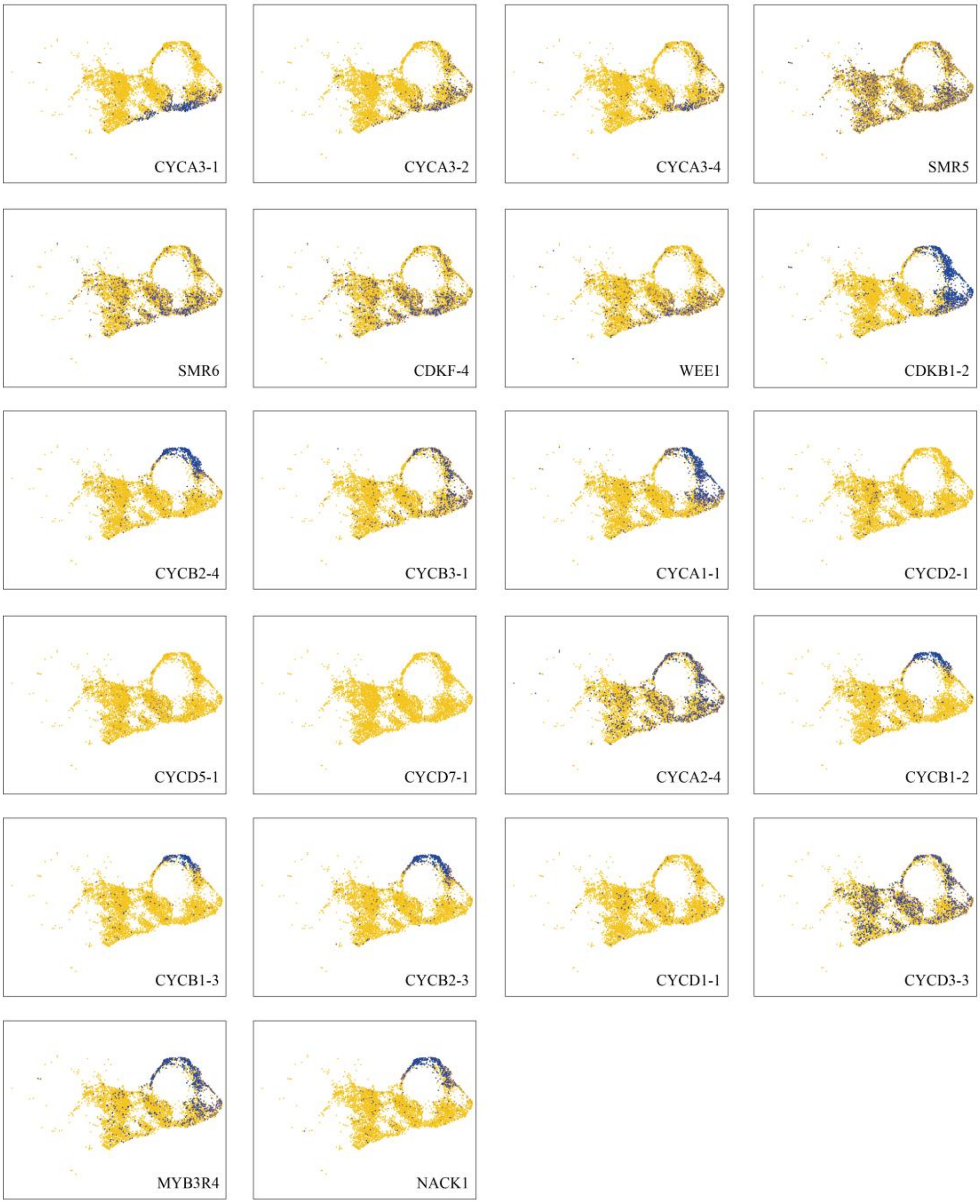

Supplemental Figure S8. Expression pattern of additional cell cycle markers.
UMAP plot with the normalized expression of the cell cycle marker gene (blue = high, yellow = low).

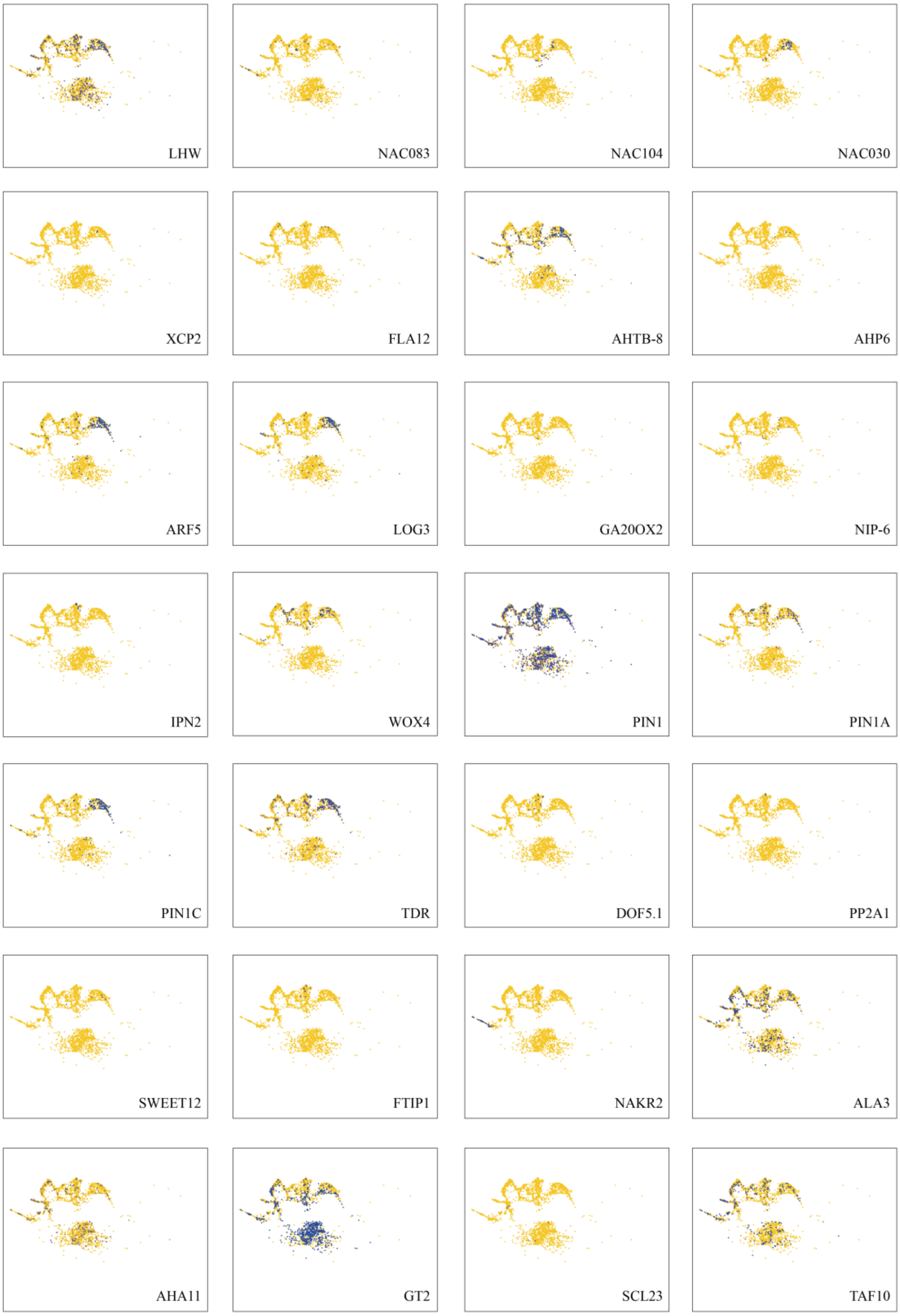

Supplemental Figure S9. Expression pattern of additional vascular cell populations markers. UMAP plot with the normalized expression of the vascular cell marker gene (blue = high, yellow = low).
